# Rapid identification of carbapenem-resistant *Klebsiella pneumoniae* based on matrix-assisted laser desorption ionization time-of-flight mass spectrometry and an artificial neural network model

**DOI:** 10.1186/s12929-023-00918-2

**Published:** 2023-04-17

**Authors:** Yu-Ming Zhang, Mei-Fen Tsao, Ching-Yu Chang, Kuan-Ting Lin, Joseph Jordan Keller, Hsiu-Chen Lin

**Affiliations:** 1 School of Medicine, College of Medicine, Taipei Medical University, Taipei, Taiwan; 2 Department of Laboratory Medicine, Taipei Medical University Hospital, Taipei, Taiwan; 3 Department of Business Administration, National Taiwan University, Taipei, Taiwan; 4 Western Michigan University Homer Stryker M.D. School of Medicine, Department of Psychiatry, Kalamazoo, MI USA; 5 Department of Clinical Pathology, Taipei Medical University Hospital, Taipei, Taiwan; 6 Department of Pediatrics, School of Medicine, College of Medicine, Taipei Medical University, No. 250, Wu-Hsing St, Taipei, 11031 Taiwan

**Keywords:** Carbapenem-resistant *Klebsiella pneumoniae*, MALDI-TOF MS, Machine learning, Artificial neural network, Antimicrobial resistance, Feature selection

## Abstract

**Background:**

Carbapenem-resistant *Klebsiella pneumoniae* (CRKP) is a clinically critical pathogen that causes severe infection. Due to improper antibiotic administration, the prevalence of CRKP infection has been increasing considerably. In recent years, the utilization of matrix-assisted laser desorption ionization time-of-flight mass spectrometry (MALDI-TOF MS) has enabled the identification of bacterial isolates at the families and species level. Moreover, machine learning (ML) classifiers based on MALDI-TOF MS have been recently considered a novel method to detect clinical antimicrobial-resistant pathogens.

**Methods:**

A total of 2683 isolates (369 CRKP cases and 2314 carbapenem-susceptible *Klebsiella pneumoniae* [CSKP]) collected in the clinical laboratories of Taipei Medical University Hospital (TMUH) were included in this study, and 80% of data was split into the training data set that were submitted for the ML model. The remaining 20% of data was used as the independent data set for external validation. In this study, we established an artificial neural network (ANN) model to analyze all potential peaks on mass spectrum simultaneously.

**Results:**

Our artificial neural network model for detecting CRKP isolates showed the best performance of area under the receiver operating characteristic curve (AUROC = 0.91) and of area under precision–recall curve (AUPRC = 0.90). Furthermore, we proposed the top 15 potential biomarkers in probable CRKP isolates at 2480, 4967, 12,362, 12,506, 12,855, 14,790, 15,730, 16,176, 16,218, 16,758, 16,919, 17,091, 18,142, 18,998, and 19,095 Da.

**Conclusions:**

Compared with the prior MALDI-TOF and machine learning studies of CRKP, the amount of data in our study was more sufficient and allowing us to conduct external validation. With better generalization abilities, our artificial neural network model can serve as a reliable screening tool for CRKP isolates in clinical practice. Integrating our model into the current workflow of clinical laboratories can assist the rapid identification of CRKP before the completion of traditional antimicrobial susceptibility testing. The combination of MADLI-TOF MS and machine learning techniques can support physicians in selecting suitable antibiotics, which has the potential to enhance the patients’ outcomes and lower the prevalence of antimicrobial resistance.

## Background

Carbapenem-resistant *Klebsiella pneumoniae* (CRKP) was rated as one of the most critical pathogens in the list of antibiotic-resistant pathogens that pose the greatest threat to human health released by the World Health Organization in February 2017 [1]. CRKP is resistant to not only carbapenems but also multiple antibiotics. Therefore, the antibiotics available to treat CRKP infection include only polymyxins, tigecycline, aminoglycosides, and fosfomycin; However, certain antibiotics had been associated with potential toxicity concerns, such as polymyxins which had been linked to the development of nephrotoxicity and neurotoxicity, while aminoglycosides were known to have side effects including nephrotoxicity and ototoxicity. In addition, concerns had been raised regarding the efficacy of some of these antibiotics, such as the low serum concentrations of tigecycline, which rendered it unsuitable for treating patients with CRKP bacteremia [2]. A large systematic review published in *The Lancet* in February 2022 demonstrated the severities of the global burden of bacterial antimicrobial resistance (AMR) [3]. Moreover, this review indicated that *K. pneumoniae* is among the six leading pathogens associated with AMR, resulting in over 600,000 deaths in 2019, and particularly causes lower respiratory and thorax, bloodstream, and intra-abdominal infections. Another systematic review included 62 studies and reported that the pooled mortality rate was 42.14% in 2462 patients with CRKP infection but 21.16% in those with carbapenem-susceptible *K. pneumoniae* (CSKP) infection. In addition, this review reported that mortality rates in North America, South America, Europe, and Asia were 33.24%, 46.71%, 50.06%, and 44.82%, respectively, indicating that CRKP is already a global problem that needs to be addressed urgently [4]. In terms of healthcare costs, Li et al. [5] further demonstrated that patients with CRKP infection had a longer hospitalization duration and a higher proportion of intensive care unit admissions (70.7% vs. 17.7%), which had posed a large burden in infection control measures in recent years.

Because of the high AMR of CRKP, the growing rate of inappropriate empiric antibiotic therapy and delayed diagnosis has become a crucial problem. Thus, a rapid detection method for carbapenem resistance is urgently required. Disk diffusion and broth dilution tests, which are used to determine the minimum inhibitory concentration (MIC), are the most commonly employed traditional methods for antimicrobial susceptibility testing (AST) in accordance with international guidelines. An additional day after the culturing of bacteria is required to determine AMR. Reverse transcription-polymerase chain reaction (RT-PCR) is another rapid, accurate, and reliable method that can be used to detect the expression levels of AMR genes. However, the cost of RT-PCR is considerably high and can only be used to detect known genes which were not the synonyms of phenotypic resistance in bacteria [6]. In contrast, the target of MALDI-TOF were expressed peptides or proteins from the specific bacteria, which contributed to the AMR directly. A review study represented that matrix-assisted laser desorption ionization time-of-flight mass spectrometry (MALDI-TOF MS) had been widely used in clinical microbiology laboratories and is a rapid, low-cost tool for accurately identifying microorganisms and even the AMR [7]. Compared with the traditional methods, the use of MALDI-TOF MS for AMR detection reduced the 30-day mortality of patients with bloodstream infection, length of hospital stay, and total hospital cost for infection control [8].

MALDI-TOF MS is a promising tool for future clinical AMR detection and has been exhibited in previous studies on different bacteria such as MRSA [9]. However, the potential genetic mutations in bacterial regulators and adaptations to growth environments could cause modifications in protein expression, leading to peak shifts with variable intervals on the MALDI-TOF MS signals [10]. The mass shift effects had presented a challenge in the preprocessing of MALDI-TOF MS data. Moreover, a systematic review had surveyed 36 studies about machine learning for detecting AMR on MALDI-TOF MS data published before 31 January 2020 and indicated the major limitations in these studies, such as small sample sizes, wide genetic diversity of certain pathogens and lack of external validation [11]. Addressing these limitations, several subsequent researches increased their sample sizes, collected data from multiple departments and conducted external validations to improve the reliability and accuracy of the MALDI-TOF MS models for clinical AMR detection [9, 12, 13].

In this study, we established a new scheme to classify CRKP and CSKP by using machine learning (ML) techniques. A methodological review had reported that artificial neural network (ANN) models were frequently employed in medical researches and had above-average accuracy in most cases [14]. Furthermore, compared to other models such as decision tree, random forest, support vector machine, ANN models offer more flexibility in terms of architecture-modifying and can be fine-tuned with various machine learning techniques such as early stopping, weight decay and dropout layer, leading to decreased overfitting effect and enhanced generalization abilities. We concatenated MALDI-TOF MS data and AST results, then constructing an ANN prediction model. In our new scheme, we were able to utilize the neural network model to analyze MALDI-TOF MS data immediately after obtaining the colony culture, and thereby aided physicians in making prompt and effective choices of antimicrobial drugs for *K. pneumoniae* infection.

## Materials and methods

### Data source

MALDI-TOF MS and AST data were collected from the clinical microbiology laboratories of Taipei Medical University Hospital (TMUH) from April 2018 to March 2022. Because all personal information was encrypted, this study was exempted from the requirement of written informed consent from patients. This study was approved by the Joint Institutional Review Board of Taipei Medical University (TMUH-JIRB reference number: N202207046). In TMUH, BD Phoenix M50 (Becton, Dickinson and Company, Maryland, USA) is used to analyze the AST of clinical isolates. All clinical isolates were collected from urine, sputum, blood, swabs from pus, wound, vagina, and other clinical specimens. According to the Clinical Laboratory Standards Institute M100 guidelines, *K. pneumoniae* isolates derived from clinical samples and exhibited resistance to any carbapenem antibiotics (ertapenem, imipenem, doripenem, and meropenem) were classified as CRKP. The other *K. pneumoniae* isolates were defined as CSKP.

### Identification of *K. pneumoniae* through MALDI-TOF MS

We used streaking method from collecting various specimen (Table 1) at agar plate (BAP, EMB, PEA and chocolate agar what agar?), and put them in 5% CO_2_ incubator to incubate bacteria according to Clinical Microbiology Procedure Handbook 4th ed. After the culturing of clinical specimens, we isolated single colonies from the and smeared them onto spots in the target plate of MALDI-TOF MS. MALDI-TOF MS samples were prepared in accordance with the manufacturer’s instructions (Bruker Daltonik, Bremen, Germany). Briefly, 1 µL of 70% formic acid was overlaid on the spots, and the target plate was dried at room temperature. Subsequently, 1 µL of α-cyano-4-hydroxycinnamic acid (10 mg/mL) was added to the matrix solution (50% acetonitrile) and 2.5% trifluoroacetic acid was added to the sample spot. Then, the sample matrix was dried at room temperature again before performing MADLI-TOF MS. MALDI-TOF MS was conducted using the MALDI-TOF mass spectrometer (Microflex LT, Bruker Daltonik, Germany). The spectrum was analyzed using MALDI Biotyper Compass software version 4.1. For MADLI-TOF MS, we used the settings recommended by the manufacturer. Each mass spectrum consisted of 240 laser shots obtained in 40-shot steps (linear positive mode; accelerating voltage, + 20 kV; and nitrogen laser frequency, 60 Hz). The calibration of Bruker MADLI-TOF MS was performed using the bacterial test standard prior to the analysis of clinical samples to ensure the validity of the identification of *K. pneumoniae*. We followed the instructions from the manufacturer, only using the isolates whose identification scores were higher than 2 as analytes in our study. The average identification score and standard deviation of these *K. pneumoniae* isolates were 2.39 ± 0.11. Moreover, in this study, the *K. pneumoniae* isolates with incomplete AST or MALDI-TOF MS data were excluded and we did not use machine learning techniques such as over-sampling method to make copies of the CRKP data.

**Table 1:** Demographics of clinical *Klebsiella pneumoniae* isolates sample demographics (univariate analysis)

	CRKP, n (%) (N = 369)	CSKP, n (%) (N = 2314)	P-value
Specimen collection			
Urine	200 (54.2%)	1134 (49.0%)	0.016
Sputum	63 (17.1%)	458 (19.8%)	
Blood	31 (8.4%)	153 (6.6%)	
Pus	26 (7.1%)	283 (12.2%)	
Wound	17 (4.6%)	80 (3.5%)	
Vagina	4 (1.1%)	53 (2.3%)	
Others	28 (7.6%)	153 (6.6%)	
Location of sampling			
Ward	160 (43.4%)	738 (31.9%)	< 0.0001
ER	72 (19.5%)	613 (26.5%)	
OPD	55 (14.9%)	552 (23.9%)	
ICU	33 (8.9%)	249 (10.8%)	
Unspecified	49 (13.3%)	162 (7.0%)	

*CRKP* carbapenem-resistant *Klebsiella pneumoniae*, *CSKP *carbapenem-sensitive *Klebsiella pneumoniae*, *ER *emergency, *ICU* intensive care unit, *OPD* outpatient department, in chi-square Test with α = 0.05

### MALDI-TOF MS data preprocessing

Raw data of mass spectrum were first preprocessed using the R package MaldiQuant v.1.21 and MALDIquantForeign v0.13 [15, 16]. Raw data of intensity from mass spectrum were preprocessed through square-root transformation, smoothed using the Savitzky–Golay filter, and subjected to baseline reduction by using the sensitive nonlinear iterative peak clipping algorithm. After the preprocessing of data in R, we performed data preprocessing and model construction in Python v3.7 as follows. First, we trimmed the spectra in the 2000 to 20,000-Da range and assigned a zero value for the missing intensity data to prevent errors in the ML model. To reduce the shift effect of the mass spectrums, we used the binning method that has been employed by several MALDI-TOF studies to determine the mean intensity of the spectrum in a specific binning size, which mostly ranges from 3 to 20 Da [12, 13]. However, we did not observe a considerable benefit of increasing the binning size to more than 1 Da in our study. We presumed that numerous computing units in our neural network models are capable of handling the deviation in the mass spectrum. The averaging of the intensity of the spectrum may result in the loss of some information from the mass spectrum, thereby reducing the efficacy of our model. Thus, for the mass spectrum data of each isolate, we considered each 1 Da as one feature, resulting in a total of 18,000 vectors from 2000 to 20,000 Da.

We used “StandardScaler” in the Python Scikit-learn package to transform the intensity to Z score in accordance with each feature; the loss function of the ML model converged rapidly toward the minima, resulting in a better outcome. Next, we used the AST labels (CRKP and CSKP) to divide MADLI-TOF MS data into two groups and set a minimum threshold of intensity to obtain a meaningful peak, which may suggest the characteristics of CRKP and CSKP isolates. Then, we calculated the ratios of meaningful peaks respectively in the CRKP and CSKP groups for each vector. To prevent our model from “curse of dimensionality”, which can reduce the efficacy of the ML model [17], we used the interquartile range (IQR) based on ratios of meaningful peaks in the two groups to classify each vector into different quarters (Q1, Q2, Q3, Q4). The best outcome of models we achieved in our study was via including vectors which were rated above Q3 (> 75%, 4500/18,000). Finally, we used these 4500 vectors as features to establish our ML model.

### Construction of the ANN model

**Fig. 1:**
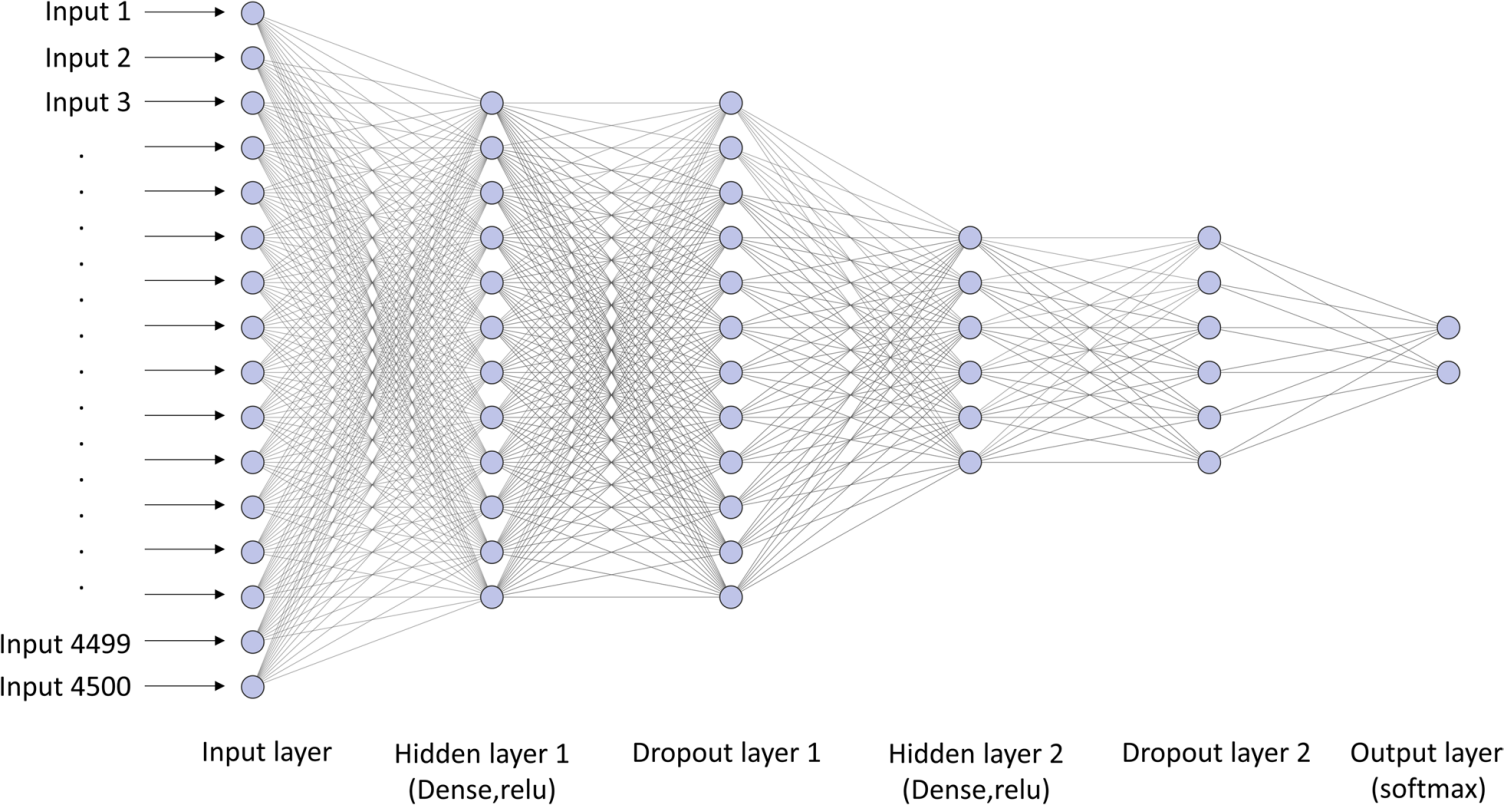
Architecture of our neural network model for identifying CRKP and CSKP isolates

We constructed the ANN model by using Python v3.7. Before the model training process, all data were shuffled (random state value = 20) and split into 80% training and 20% independent validation data sets, stratified by AST labels to ensure the absence of imbalance in both data sets. This independent validation data set was not used for the model training process. We employed this data set only to externally evaluate the performance of our model. In this study, we constructed an ANN model to predict the resistance of *K. pneumoniae* to carbapenems. This model included 4500 features selected from Q3 based on the calculated IQR. The established neural network model included one input layer, two dense layers with the rectified linear unit (ReLU) activation function, two dropout layers, and one output layer with the softmax activation function (Fig. 1). The input layer consisted of 4500 vectors selected using the IQR method, and the output layer with the softmax activation function yielded two prediction values for both classes (CRKP and CSKP).

The illustration was generated using this web tool: http://alexlenail.me/NN-SVG/index.html.

### Model evaluation

During the model training process, we used tenfold cross validation to evaluate the accuracy of our model and simultaneously modified weights in the neurons of the model to minimize the loss of function. This process helped in evaluating the model performance and obtaining a better outcome. After the completion of the training process, we shuffled the independent validation data set (random state value = 1) and chose a balanced number of CSKP and CRKP isolates from the above data set to perform external validation, verifying the efficacy of our model with, sensitivity, specificity, F1-score, accuracy, area under the receiver operating characteristic curve (AUROC) and of area under precision–recall curve (AUPRC).

### Statistical analysis

Statistical analyses were performed using the SPSS statistics program version 19 (IBM Corporation: New York, NY, USA). The chi-squared test was applied to compare nonparametric frequency of categorical variables between the two groups. A two-sided *p* value of < 0.05 indicated statistical significance. Receiver operating characteristic (ROC) curve was used to evaluate the performance of machine learning models and precision–recall curve (PRC) was suitable for evaluating the performance of models when the data distribution was imbalanced. The efficacy of our prediction model was presented as sensitivity, specificity, F1-score, accuracy as follows: Sensitivity = [True Positives (CRKP)]/[True Positives (CRKP) + False Negatives]; Specificity = [True Negatives (CSKP)]/[True Negatives (CSKP) + False Positives]; Accuracy = [True Positives (CRKP) + True negative (CSKP)]/[True Positives (CRKP) + True Negatives (CSKP) + False Positives + False Negatives].

## Results

### Overview of AST and MALDI-TOF MS data

After matching AST data with available MADLI-TOF MS data, we included 2683 isolates (369 CRKP and 2314 CSKP) in this study. The collection of the specimen and location of the sampling significantly differed between the CSKP and CRKP isolates (Table 1).

### Model performance for the detection of CRKP and CSKP

**Fig. 2:**
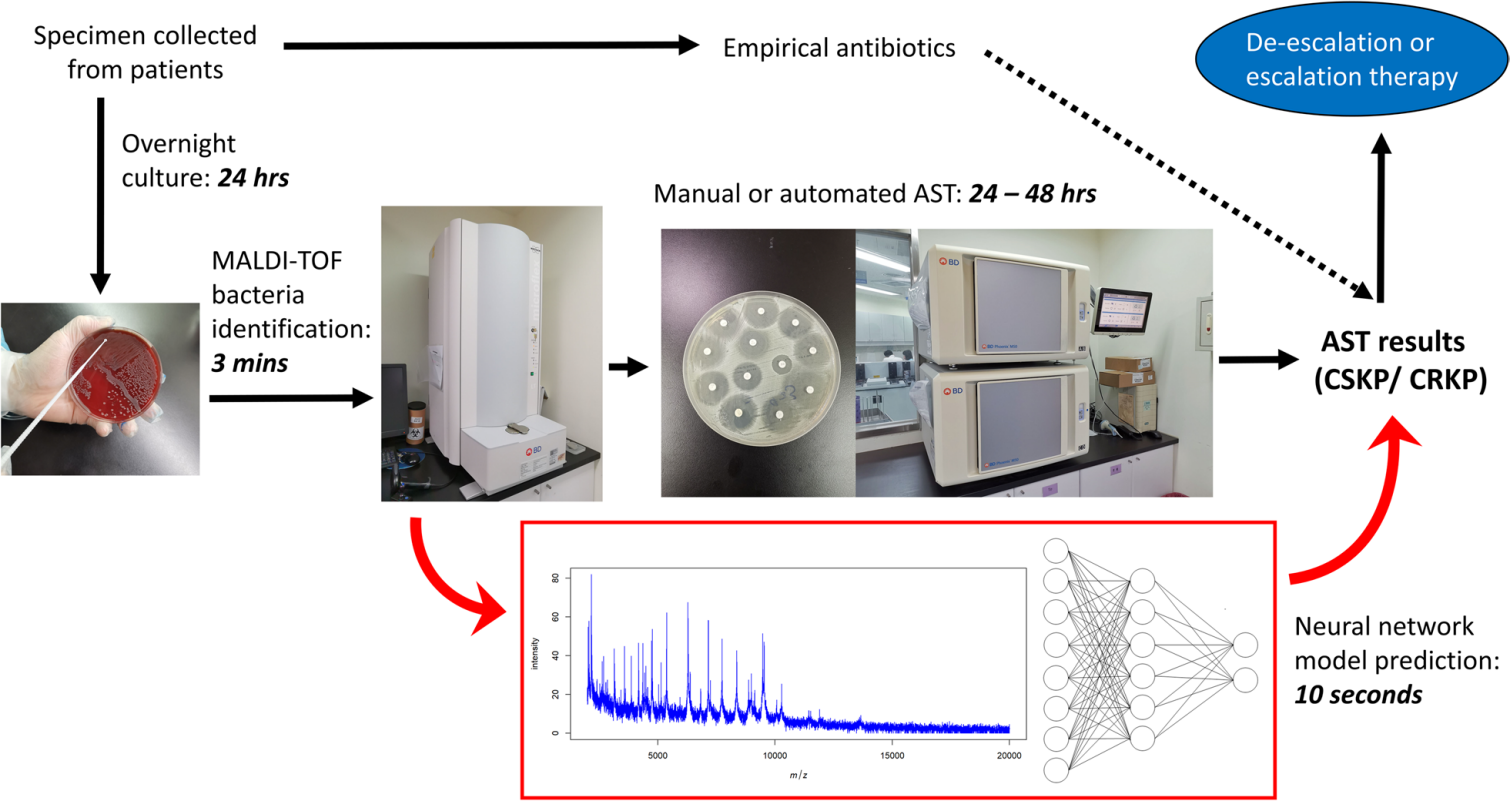
The figure shows the current workflow in clinical laboratories of TMUH involved collecting specimens from the bedside to obtain an AST report, which takes at least 72 h. Clinicians can then determine whether they need to switch from empiric antibiotics to a more appropriate option. Integrating our model into this workflow at the framed part, we can screen the CRKP isolates and provide clinicians with guidance before the AST reports are available. The new scheme can shorten the turnover time of detecting CRKP and further benefit physicians who require AST results for specific antibiotics that are not part of the routine

In the traditional workflow, after culturing, bacterial identification should be performed first using MALDI-TOF. Then, AST would require 24 to 48 h. In clinical practice, at least 3 to 4 days would be required for the identification of CRKP isolates. By using our neural network model, we obtained the results at least 1 day earlier than traditional AST. Thus, this model has the potential to aid in the screening of CRKP and assists clinicians in the selection of appropriate antibiotics promptly. In this study, we propose a new scheme for the detection of CRKP isolates (Fig. 2). Figure 2 shows the current workflow in clinical laboratories of TMUH involved collecting specimens from the bedside to obtain an AST report, which takes at least 72 h. Clinicians can then determine whether they need to switch from empiric antibiotics to a more appropriate option. Integrating our model into this workflow at the framed part, we can screen the CRKP isolates and provide clinicians with guidance before the AST reports are available. The new scheme can shorten the turnover time of detecting CRKP and further benefit physicians who require AST results for specific antibiotics that are not part of the routine.

**Fig. 3:**
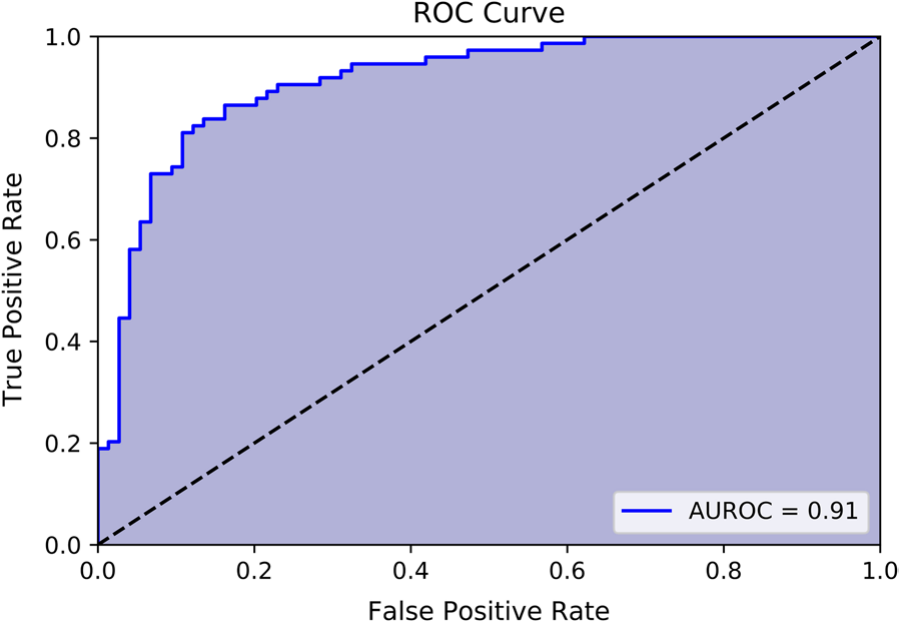
The area under the receiver operating characteristic curve (AUROC) represents the performance of the predictive ANN model for identifying CRKP isolates. In external validation with a balanced number of CSKP and CRKP isolates, the AUROC is 0.91

**Fig. 4:**
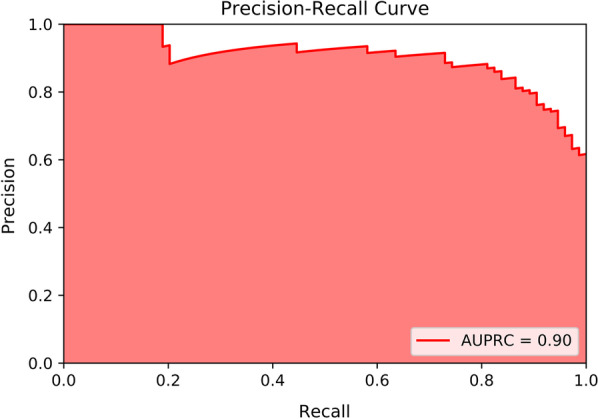
The area under the precision–recall curve (AUPRC) is used to assess the efficacy of machine learning models when the training or validation dataset is imbalanced. An AUPRC value of 0.90 is obtained in the external validation of the ANN model with an equal number of CSKP and CRKP isolates. This indicates that the model is robust despite the imbalanced distribution of the dataset

We established an ANN model and used 80% of total data as the training data set (n = 2146), enabling our model to distinguish CRKP from CSKP. Then we verified the efficacy of our model with sensitivity, specificity, F1-score, accuracy, AUROC and AUPRC. The remaining 20% of the total data were regarded as the independent validation data set and we chose a balanced number of CSKP and CRKP isolates (N = 74, 74) from the data set to perform external validation. In the external validation, we used the sensitivity, specificity, F1-score, accuracy, AUROC and AUPRC to measure the performance of our model. Our neural network model had a sensitivity of 0.84, a specificity of 0.84, a F1-score of 0.84, an accuracy of 0.84. In external validation with a balanced number of CSKP and CRKP isolates, the AUROC is 0.91 (Fig. 3). The area under the precision–recall curve (AUPRC) is used to assess the efficacy of machine learning models when the training or validation dataset is imbalanced. An AUPRC value of 0.90 (Fig. 4) is obtained in the external validation of the ANN model with an equal number of CSKP and CRKP isolates. This indicates that the model is robust despite the imbalanced distribution of the dataset.

### Analysis of significant peaks by using Shap values

**Fig. 5:**
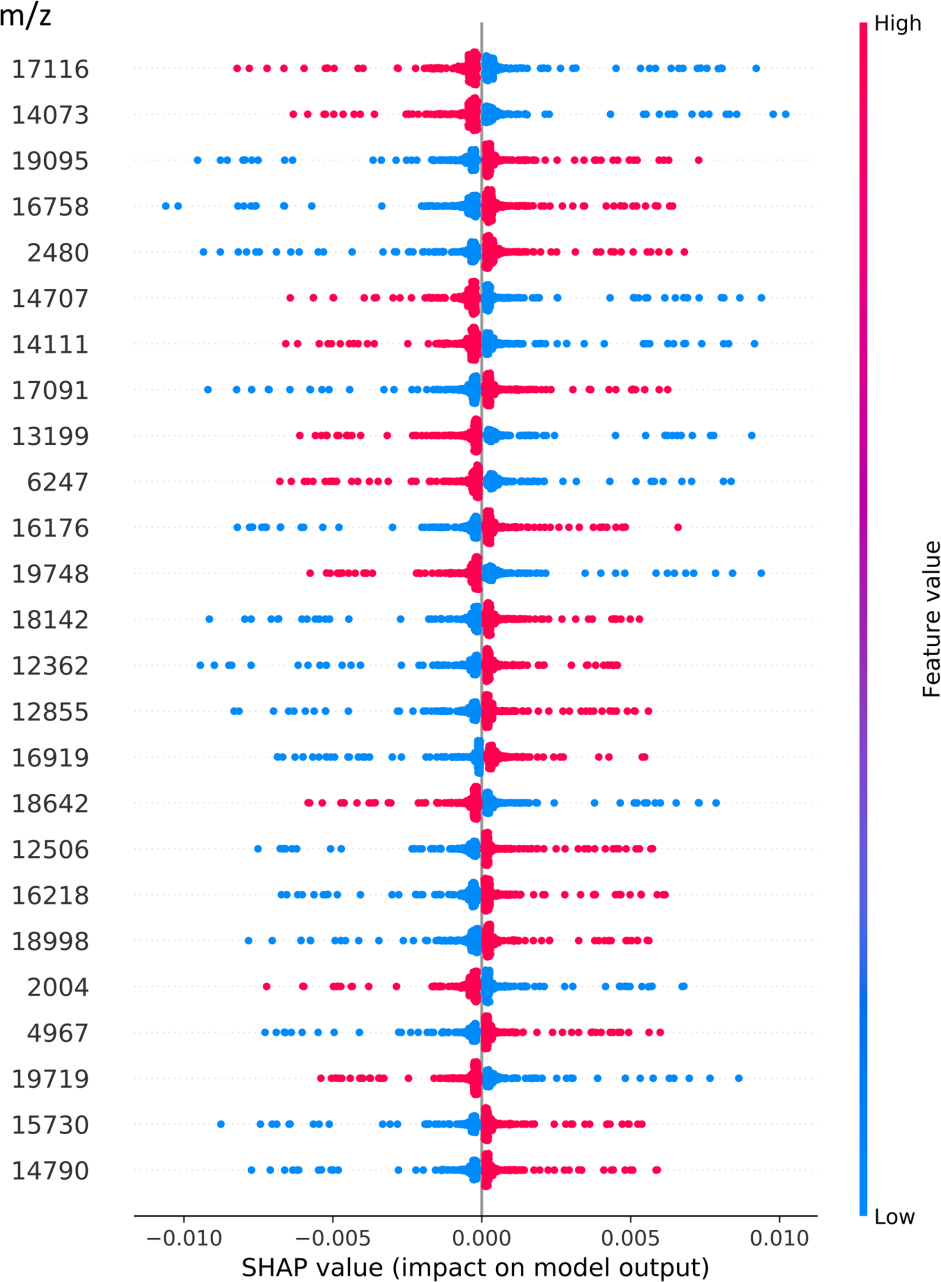
Shap value, an explainable AI method, is used to evaluate the impact of each feature in models, increasing transparency and interpretability of machine learning models. The accompanying illustration displays the shap values of the leading 25 features in our ANN model and may provide valuable insights for future researches

We further analyzed our ANN model and identified potential biomarkers using the Shap package in Python v3.7 to summarize the importance of specific peaks. As presented in Fig. 5, the red color represents isolates with specific peaks in the mass spectrum and the blue color represents isolates without specific peaks. Shap value, an explainable AI method, is used to evaluate the impact of each feature in models, increasing transparency and interpretability of machine learning models. The accompanying illustration displays the shap values of the leading 25 features in our ANN model and may provide valuable insights for future researches. The efficiency to predict whether an *K. pneumoniae* isolate is resistant to carbapenems increases as the distance away from the zero point of Shap values increases. In this study, we proposed the top 15 potential biomarkers in probable CRKP isolates at 2480, 4967, 12,362, 12,506, 12,855, 14,790, 15,730, 16,176, 16,218, 16,758, 16,919, 17,091, 18,142, 18,998, and 19,095 Da.

## Discussion

In the current workflow, at the first instance, physicians determined suitable empiric antibiotic therapy based on the patient’s medical history, current illness and severity. We developed an ANN model by using MALDI-TOF MS data and AST labels (CRKP/CSKP). Compared with traditional AST, this low-cost ML model could rapidly and accurately distinguish CRKP from CSKP despite the use of different specimens. Early and rapid detection of CRKP in clinical isolates can help physicians in providing appropriate empiric antibiotics to patients. In studies identifying bacteria with AMR, ML models based on MALDI-TOF MS data have been widely used, and the most discussed bacteria was *Staphylococcus aureus* [18–20]. However, only a few studies using ML models have focused on CRKP; There were two studies about CRKP identification based on MALDI-TOF MS and ML techniques published on February 2020 and July 2022 [21, 22]. Nevertheless, the two studies did not provide information on whether they had split an independent validation data set from their whole database to perform external validation. There was a doubt of that their models might have overfitted to their own training data set if without performing external validations. The overfitting effect was particularly harmful to the performance of models when the size of the training data set was small. In the two studies, the total sample sizes were 95 and 171, respectively. Although the accuracy of the models in those studies was over 90%, the performance and generalization abilities of their models might be limited in the real world. The lack of external validation was widely noted in many studies. A systematic review published in October 2020 indicated that only approximately 11% (4/36) of MALDI-TOF machine learning studies performed external validation [11]. In our study, the sample size of the data set was 20 times more than the two previous CRKP studies on average. Moreover, we performed external validation by using an independent validation data set to examine the performance and robustness of our model in the real world, which was more reliable than the prior CRKP studies.

The potential biomarkers of CRKP isolates identified in our study are different from those reported in the previous two studies. This difference can be attributable to the the local genetic diversity and lack of the generalization abilities of the models from previous studies. Moreover, in our study, we performed both AST and MALDI-TOF MS on the Bruker system in the clinical laboratory of TMUH, whereas the two CRKP studies performed AST and MALDI-TOF MS on a VITEK system. Differences in the composition of the matrix, settings of laser frequency, and processes might reduce comparability between the two systems.

Previous studies examining the specific strains of *K. pneumoniae* or *K. pneumoniae* carbapenemase*-*producing *K. pneumoniae* (KPC-Kp) have reported different findings for the mass spectrum. Centonze et al. [23] reported that the peak of 11,109 Da was widely detected in KPC-Kp and indicated that this peak can be beneficial in detecting KPC-Kp. Figueroa-Espinos et al. [24] reported that the peak of 11,109 Da only existed in 32% of KPC-Kp and observed another peak of 28,544 Da, where the *blaKPC-2* gene was embedded. However, Huang et al. [25] demonstrated that no peak of 11,109 Da was detected in their KPC-Kp isolates, and all of them were ST11, the dominant clone in China. They analyzed 235 isolates and found a peak of 4521 Da in *blaKPC-2*-positive isolates but not in *blaKPC-2*-negative isolates. Variable peaks on MADLI-TOF MS may be observed due to the different local genetic diversity of *K. pneumoniae* isolates. On account of the genetic variability and diverse mechanisms of antimicrobial resistance in CRKP isolates, we could not analyze the distribution of each specific strain in this study.

### Limitations

Our study has some limitations. First, we did not examine the expression of genes associated with carbapenem resistance in *K. pneumoniae*, including *ST11*, *KPC*, *VIM*, *IMP*, *NDM*, and *OXA-48* through PCR in our study. Gene identification is not required in clinical practice. Second, our AST and MALDI-TOF MS data set of *Klebsiella pneumoniae* were obtained from the TMUH clinical laboratory. We utilized all eligible *K. pneumoniae* isolates to establish our model, but we did not investigate the distribution of different *K. pneumoniae* strains. Therefore, the efficacy of our model in predicting *K. pneumoniae* isolates from other laboratories or countries needs to be examined in future studies. More data from different hospitals, regions and even countries are required for training ML models to achieve a greater ability of generalization.

## Conclusion

In this study, we established a robust ANN model by using AST and MALDI-TOF MS data to accurately identify CRKP with an AUROC of 0.91 and an AUPRC of 0.90. Our study differed from previous literatures that utilized MALDI-TOF MS and machine learning techniques to identify CRKP isolates, as we had a more comprehensive dataset and divided the entire database into a training set and an independent validation set to further validate our model. Our ANN model demonstrated better generalization abilities, making it a dependable screening tool for CRKP isolates in clinical settings. Integrating our model into the current workflow of clinical laboratories could assist in the rapid identification of CRKP prior to completing traditional antimicrobial susceptibility testing. Our new approach, combined with traditional antimicrobial susceptibility testing, can aid in selecting appropriate antibiotics, which has the potential to improve patients’ outcomes and decrease the prevalence of antimicrobial resistance.

## Data Availability

The de-identified data supporting the conclusions of our research are available from the corresponding authors, without undue reservation, to qualified researchers.

## Abbreviations

CRKP: Carbapenem-resistant *Klebsiella pneumoniae*
MALDI-TOF MS: Matrix-assisted laser desorption ionization time-of-flight mass spectrometry
ML: Machine learning
TMUH: Taipei Medical University Hospital
ANN: Artificial neural network
AUROC: Area under the receiver operating characteristic curve
AUPRC: Area under the precision–recall curve
ReLU: Rectified linear unit
AMR: Antimicrobial resistance
CSKP: Carbapenem-susceptible *K. pneumoniae*
MIC: Minimum inhibitory concentration
AST: Antimicrobial susceptibility testing
RT-PCR: Reverse transcription–polymerase chain reaction
M/Z: Mass-to-charge ratio
IQR: Interquartile range
KPC-Kp: *K. pneumoniae* carbapenemase-producing *K. pneumoniae*
